# Increased Left Putamen Volume Correlates With Pain in Ankylosing Spondylitis Patients

**DOI:** 10.3389/fneur.2020.607646

**Published:** 2020-11-30

**Authors:** Kelei Hua, Peijun Wang, Zhihong Lan, Meng Li, Wenkai Zhao, Tianyue Wang, Shumei Li, Xiaofen Ma, Chao Li, Shishun Fu, Yi Yin, Ping Liu, Jin Fang, Tianwang Li, Guihua Jiang

**Affiliations:** ^1^The Second School of Clinical Medicine, Southern Medical University, Guangzhou, China; ^2^Department of Medical Imaging, Guangdong Second Provincial General Hospital, Guangzhou, China; ^3^Department of Medical Imaging, Traditional Chinese Medicine-Integrated Hospital of Southern Medical University, Guangzhou, China; ^4^Department of Rheumatology and Immunology, Guangdong Second Provincial General Hospital, Guangzhou, China

**Keywords:** ankylosing spondylitis, gray matter volume, voxel-based morphometry, pain, disease duration

## Abstract

Ankylosing spondylitis (AS) mainly affects the axial skeleton and is an important factor leading to chronic lower back pain in young individuals. However, few studies have explored alterations of brain gray matter volume in AS patients. The purpose of the present study was to describe brain gray matter abnormalities associated with AS pain. A total of 61 AS patients and 52 healthy controls (HCs) were included in this study. Using voxel-based morphometrics, we detected abnormal gray matter volume in AS patients. Based on the voxel-wise analysis, the gray matter volume in the left putamen of the AS group was increased significantly compared with that of the HC group. In addition, we found that the gray matter volume of the left putamen was positively correlated with the duration of AS and total back pain scores, whereas it was not significantly correlated with Bath Ankylosing Spondylitis Disease Activity Index scores, C-reactive protein, or erythrocyte sedimentation rate in AS patients. Taken together, our findings improve our understanding of the neural substrates of pain in AS and provide evidence of AS-related neurological impairment. Hence, further investigation of the pathophysiology of the left putamen in AS is warranted.

## Introduction

Ankylosing spondylitis (AS) is a chronic inflammatory rheumatic disease with unknown etiology that mainly affects the axial skeleton and is an important factor leading to chronic lower back pain in young individuals (1, 2). Ranganathan et al. (3) summarized the current knowledge of the pathogenesis of AS, including genetic risk association, HLA-B27-mediated pathology, perturbations in antigen presentation pathways and contributions of type 3 immune responses. Among the genes associated with AS, HLA-B27 confers the greatest risk and is present in 85–90% of patients (4). However, lower back pain is one of the important factors leading to unsatisfactory treatment efficacy, poor quality of life, and even disability in AS patients (5). Similar to the symptoms of patients with other forms of chronic pain, AS patients who are experiencing pain manifest both functional and structural brain alterations (6, 7). The development of functional magnetic resonance imaging (MRI) technology has enabled researchers to detect abnormalities in neural activity and brain structures and has provided valuable insights into brain function and structure (8, 9). Accumulated findings from brain functional and structural imaging studies have demonstrated that chronic pain may induce neurological impairment, such as chronic back pain (10), trigeminal neuralgia (11), and AS pain (12, 13). These results highlight that chronic pain can arise from a variety of different pathological factors, among which the brain mechanisms of AS pain have remained unclear.

In addition, imaging studies have linked chronic pain to specific brain structures and networks. Using the functional connectivity (FC) method, Hemington et al. (6) found that the default mode network and salience network were altered and closely related to chronic pain in AS patients. Using both amplitude of low-frequency fluctuations (ALFF) and FC methods, Li et al. (13) found that the ALFF values of the left medial frontal gyrus, right precentral gyrus, and right posterior cingulate were significantly reduced, while the ALFF values of the left cerebellum anterior lobe, left middle temporal gyrus, left postcentral gyrus, and right precuneus were significantly increased in AS patients. Additionally, the FCs of the left precuneus and left middle temporal gyrus of AS patients were closely related to Bath Ankylosing Spondylitis Disease Activity Index (BASDAI) scores, erythrocyte sedimentation rate (ESR), and C-reactive protein (CRP). However, to the best of our knowledge, few studies have focused on alterations of brain gray matter volume in AS patients. Furthermore, there has been no consensus on the precise location of functional and structural abnormalities in the brain in these previous studies, due to differences in sample sizes and image processing. Voxel-based morphometry (VBM) is an automated, unbiased approach for voxel-wise comparisons of gray or white matter between different populations via high-resolution MRI scans (14). Many studies have already demonstrated structural differences in the brains among different groups of patients using VBM technology (15–17). Using the VBM method, Wu et al. (7) found that the primary somatosensory, insular, anterior cingulate, and supplemental motor area showed cortical thinning, and that the thalamus and putamen showed hypertrophy in AS patients. Additionally, painDETECT scores were correlated with decreased gray matter volume in the primary somatosensory cortex and increased gray matter volume in the motor cortex, anterior cingulate cortex, prefrontal cortex, thalamus, and striatum in AS patients. However, the sample size of this previous study was not large, limiting its statistical power. Additionally, it remains unclear whether and how the development course of AS affects alterations in the human brain, which are essential issues for better understanding the underlying mechanisms of pain in AS patients.

Therefore, in the present study, we used VBM to explore differences in brain gray matter volume between AS patients and healthy controls (HCs). We hypothesized that there are distinctive abnormalities in brain gray matter volume associated with AS pain, and that such brain changes may provide further insight into the neural substrates of AS-related pain.

## Materials and Methods

### Subjects

This study was approved by the Ethics Committee of Guangdong Second Provincial General Hospital. All subjects signed informed consent after fully understanding the research content. A total of 52 HCs and 61 AS participants, all of whom were right-handed, were enrolled in our study. AS patients were only recruited if they met the following conditions: (1) diagnosis of active AS in line with modified New York criteria (18); (2) non-steroidal anti-inflammatory drugs (NSAIDs) were only taken at a stable dose if there was pain; (3) no biological agents were being taken during the study or at any other time; (4) no comorbidities, such as depression, anxiety, and presence of fibromyalgia and so on; and (5) reporting of an average total back pain (TBP) score of ≥ 3 (on a 10-point scale, 0 = no pain, and 10 = worst pain imaginable) over the previous week. General inclusion criteria for all study participants were as follows: (1) 16–50 years old; (2) no prior diagnosis of mental illness or neurological disease; (3) no major surgery in the past 2 years; and (4) no other MRI contraindications.

### Clinical Assessments

The clinical assessment for each AS patient included BASDAI and TBP scores. BASDAI scores were assessed by the same trained rheumatologist. The BASDAI score—which includes spinal pain, joint pain/swelling, fatigue, localized tenderness, and morning stiffness—provides a comprehensive summary of symptom severity, with a scale from 0 to 10 (i.e., 10 points indicates highest disease severity).

### MRI Scans

A 3T Philips Ingenia MR scanner, with a 32-channel NV coil, was used for MRI scans in the Department of Medical Imaging at Guangdong Second Provincial General Hospital. Fast-field echo (FFE) pulse sequences were used to obtain T1-weighted 3D high-resolution brain structural images via the following parameters: repetition time (TR) = 7.9 ms; echo time (TE) = 3.7 ms; flip angle (FA) = 8°; acquisition matrix = 256 × 256; field of view (FOV) = 256 × 256 mm^2^; slice thickness = 1.0 mm; and 185 sagittal slices.

### Data Processing

Based on SPM12 (http://www.fil.ion.ucl.ac.uk/) and Diffeomorphic Anatomical Registration Through Exponentiated Lie Algebra (DARTEL) (19), structural images were processed and analyzed via VBM. Preprocessing included segmentation of gray matter, mean template creation, spatial normalization into the MNI template space, and modulation to adjust for volumetric changes during spatial normalization. After normalization and modulation, the gray matter partitions were smoothed with an isotropic Gaussian kernel with an 8-mm full-width at half maximum (FWHM).

### Statistical Analysis

Significant differences in age and education were assessed through two-sample *t* tests, and sex composition was computed by Chi square tests between the AS and HC groups via SPSS 20.0. Two-sample *t* tests were also used to compare the gray matter volumes between AS patients and HCs. An absolute threshold mask of 0.1 was used. According to random Gaussian field theory, significant differences between the two groups of all voxels were estimated by distribution approximation, and the significance level was set as *P* < 0.05 (corrected for FWE). Partial correlations between gray matter volumes of different brain regions—which showed group differences and clinical results including BASDAI scores, TBP scores, serum CRP, ESR, and the duration of the disease—were analyzed for AS patients. Again, the significance level was set at *P* < 0.05.

## Results

### Participant Characterization

The demographics and clinical data of the participants in this study are shown in Table 1. There were no significant differences between the AS and HC groups in terms of age, gender, education, or total intracranial volume (*P* > 0.05). The mean duration of back pain in the AS group was 7.51 years.

**Table 1:** Demographic characteristics of AS patients and HCs.

**Characteristics**	**AS (*n* = 61)**	**HC (*n* = 52)**	***P*-value**
Age (years)	24.21 ± 7.06	25.75 ± 6.81	0.244^b^
Gender (male/female)	52/9	45/7	0.844^a^
Education (years)	10.30 ± 2.87	11.17 ± 3.71	0.168^b^
Total back pain	5.77 ± 1.50	N/A	–
AS duration (years)	7.51 ± 6.44	N/A	–
BASDAI	4.48 ± 1.50	N/A	–
ESR	14.66 ± 6.87	N/A	–
CRP	12.19 ± 9.17	N/A	–

*Unless otherwise indicated, data represent the means ± standard deviations. NA, not applicable*.

a *P-value was obtained using the two-tailed Chi-squared test*.

b *P-value was obtained using the two-sample, two-tailed t test*.

### Morphometry Analysis

**Figure 1:**
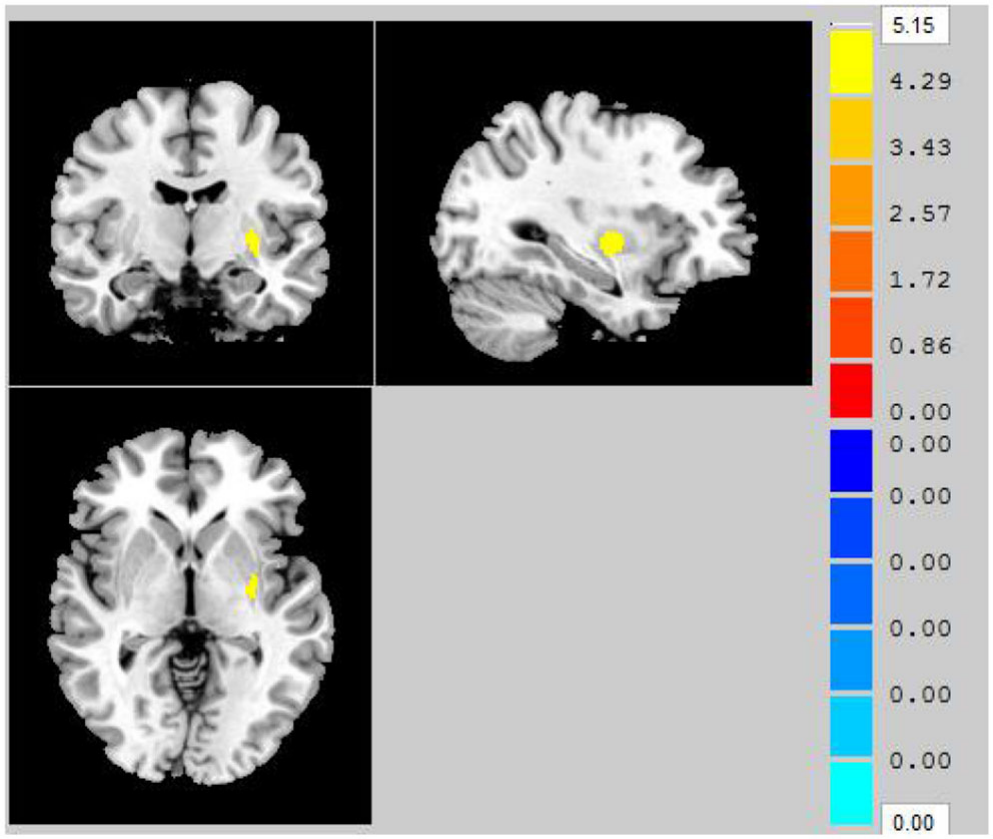
Clusters showing significantly decreased gray matter volume in AS patients compared to that in HCs (*P* < 0.05, FWE corrected).

Compared to that in the HC group, throughout the entire brain, VBM analysis revealed that one gray matter volume cluster was significantly increased in the AS group. The location of the cluster in a standard brain is shown in Figure 1, and detailed information of this brain region is provided in Table 2. Specifically, this cluster with increased gray matter volume in the AS group was located in the left putamen.

**Table 2:** Brain region showing significantly changed gray matter volume in AS patients compared to that in HCs.

**Brain area**	**MNI**				
	**Voxel size**	**x**	**y**	**z**	***T*-value**
Putamen_L	102	−31.5	−9	−1.5	5.149

### Correlational Analysis

**Figure 2:**
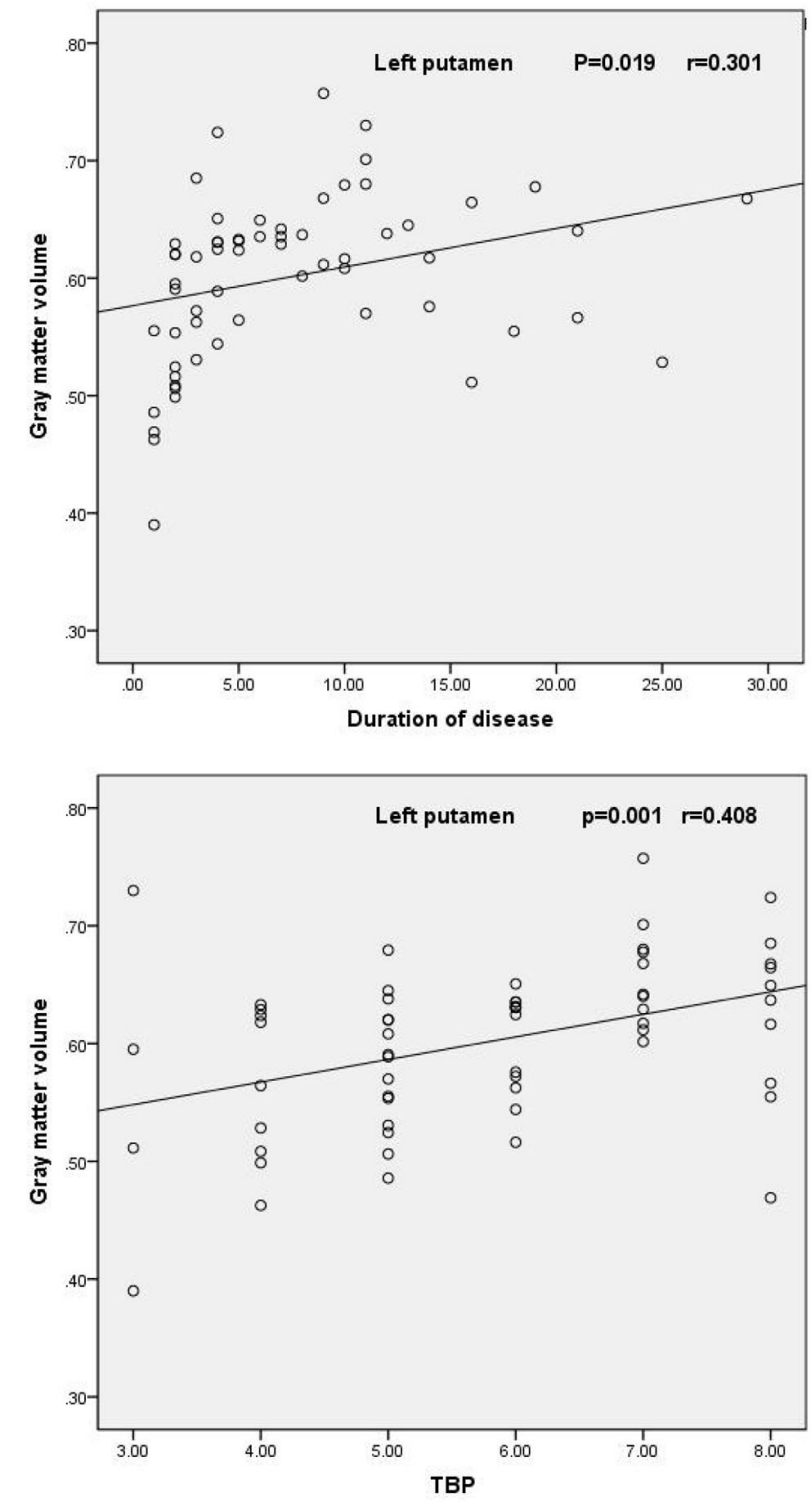
Scatter plot of the mean volume of gray matter in the left putamen as a function of the duration of AS and TBP scores.

Correlational analysis revealed that the mean gray matter volume of the left putamen was significantly positively correlated with the duration of AS (*r* = 0.301, *P* = 0.019) and TBP scores (*r* = 0.408, *P* = 0.001) (Figure 2). However, there were no other significantly positive or negative correlations between the gray matter volume of the left putamen and BASDAI scores, ESR, or CRP in AS patients.

## Discussion

Using VBM, we explored differences in gray matter volume between AS patients and HCs. Our findings showed that AS patients exhibited significantly increased gray matter volume in the left putamen compared with that in the HC group. In addition, the gray matter volume of the left putamen was positively correlated with the duration of AS and TBP scores. These findings suggest that the left putamen may be the most affected brain area that is implicated with chronic pain in AS. The altered gray matter volume in this brain area suggests that hypertrophy of the left putamen may be a potential abnormality in the central nervous system of AS patients.

The putamen, pallidum, and caudate nucleus together constitute the striatum, which is a major source of cortical and subcortical input into the basal ganglia (20). The striatum is rich in opioid receptors and contains nociceptive neurons (21–23). Additionally, using genetic transneuronal tracing analysis in adult mice, researchers have found that nociceptive neurons in the fifth layer of the spinal cord project directly into the globus pallidus, which is a structure closely related to basal ganglia circuitry and the striatum (24). Neuroanatomical studies have indicated that nociceptive neurons of the basal ganglia may be involved in the regulation of pain (25). Previous studies have demonstrated that there are structural changes in striatal gray matter in patients with various chronic pain syndromes, such as chronic lower back pain, chronic vulvar pain, or fibromyalgia (26–28). As part of the basal ganglia. The putamen has been traditionally related to motor functions (29, 30); however, it is also often activated during pain perception and is related to pain-related motor responses (31–34). Clinically, patients with lenticular (composed of the putamen and pallidum) infarctions exhibit a loss of sensation including pain (35). Therefore, the putamen and related basal ganglia circuits are critical for establishing the characteristics of pain sensation, indirectly indicating that the putamen may be related to the management of pain sensation. To test the hypothesis that the putamen contributes to pain sensation, Starr et al. (36) found that patients with putamen lesions showed reduced heat pain sensitivity and extensive reduction of pain-related brain activation. This suggests that the putamen may affect the activity of numerous brain regions related to pain perception. This result may explain why only the gray matter volume of the left putamen was increased in AS patients in our present study, and a previous study found that pain is related to a wide range of brain regions (37). Furthermore, the putamen is an important node of cortical and subcortical input into the basal ganglia (20). In addition, according to structural connectivity analysis in healthy subjects (36), the bilateral putamen activated during pain is not only associated with brain regions involved in sensorimotor processes but also associated with brain regions that play an important role in attention, emotion, and memory, which further supports our current argument. Taken together, the hypertrophy of the left putamen in AS patients in the present study suggests that patients with AS experience pain for a long period of time, which leads to increases in gray matter volume of this region. Hence, we propose a scenario that increased nociceptive input caused by inflammation of the spine may lead to hypertrophy of the left putamen.

Additionally, we found that the gray matter volume in the left putamen was positively correlated with the duration of AS and TBP scores. This correlation may indicate the longer the duration of the disease, as well as more intense pain. Furthermore, this finding may be related to the larger gray matter volume of the left putamen in AS patients. Moreover, there were no significantly positive or negative correlations between the gray matter volume of the left putamen and BASDAI scores, ESR, or CRP. BASDAI is considered as the reference standard for the evaluation of AS disease activity. And ESR and CRP are acute phase reactants of inflammation (38). The result indicates that pain in patients with AS may not always be caused by inflammation. This result might explain why pain exists in the absence of sacroiliitis on plain radiography in non-radiographic AS (39).

There were several limitations of our present study. First, as a cross-sectional study, the directionality of the relationship between AS and abnormal gray matter of the left putamen remains unclear; future longitudinal studies are needed to resolve this question. Second, because of the cross-sectional group data, we were unable to observe dynamic volumetric changes over the developmental course of AS. Future studies should address these issues through longitudinal assessment of a large sample of AS patients.

In conclusion, we used VBM to reveal increased gray matter volume of the left putamen in AS patients. We also found that the duration of AS and TBP scores was correlated with increased gray matter volume in the left putamen. These results may enhance our understanding of the neural substrates of pain in AS and provide evidence of AS-related neurological impairment. Furthermore, combined with previous research, the abnormal volume of the left putamen may be closely related to pain formation and perception in AS. Hence, further investigation of the pathophysiology of the left putamen in AS is warranted.

## Data Availability Statement

The original contributions presented in the study are included in the article/supplementary materials, further inquiries can be directed to the corresponding author/s.

## Ethics Statement

The studies involving human participants were reviewed and approved by Ethics Committee of Guangdong Second Provincial General Hospital. Written informed consent to participate in this study was provided by the participants' legal guardian/next of kin.

## Author Contributions

GJ designed the experiment. KH, PW, and ZL carried out the experiment. ML, TW, SL, and WZ collected and sorted out the data. XM, CL, SF, YY, PL, TL, and JF helped on data management and processing. KH, PW, and ZL wrote the manuscript. All authors contributed to the article and approved the submitted version.

## Conflict of Interest

The authors declare that the research was conducted in the absence of any commercial or financial relationships that could be construed as a potential conflict of interest.
